# Housing and Life Course Transitions in Later Life: The Role of Housing, Place, and Sense of Home in Periods of Uncertainty

**DOI:** 10.1093/geroni/igaa057.2001

**Published:** 2020-12-16

**Authors:** Anna Wanka, Steven Schmidt, Richard Settersten

## Abstract

Housing is central factor for health and well-being in later life. Many countries have implemented ageing in place policies, but they tend to neglect the dynamic nature and heterogeneity of the ageing process. Housing needs change as people grow older, and experience different transitions across their life courses. Studies have demonstrated relationships between housing and health and wellbeing in later life on the one hand and life transitions and health and wellbeing in later life on the other hand. However, research on life transitions in combination with objective and perceived housing in relation to indicators of good ageing is scarce. Hence, the symposium aims to explore the dynamic relationship between housing and life transitions and how this relationship impacts health, well-being, functioning, and social/neighborhood participation along the process of ageing. First, Anna Wanka and Frank Oswald investigate how older adults’ relationship to their home is interlinked with life-course transitions and social exclusion, presenting case studies from three countries. Maya Kylén explores the meaning of home and health dynamics throughout the retirement transition among the ‘younger old’ in Sweden. Kieran Walsh asks how ‘sense of home’ interrelates with risks entailed in the transitions of bereavement, dementia on-set and forced migration. Finally, Helen Barrie discusses the transition to homelessness based on the HILDA survey to identify the profile(s) of older people at risk of homelessness in Australia. Finally, Richard A. Settersten will discuss the four contributions.

